# Seven new species of the *Rhodocybe*– *Clitopilus* clade (*Entolomataceae*, *Agaricales*) from Northeast China

**DOI:** 10.3897/mycokeys.132.181204

**Published:** 2026-05-21

**Authors:** Tian-Yu Zhang, Tolgor Bau

**Affiliations:** 1 College of Mycology, Jilin Agricultural University, Changchun, China College of Mycology, Jilin Agricultural University Changchun China; 2 Key Laboratory of Edible Fungal Resources and Utilization (North), Ministry of Agriculture and Rural Affairs, Jilin Agricultural University, Changchun 130118, China Ministry of Agriculture and Rural Affairs, Jilin Agricultural University Changchun China

**Keywords:** *
Entolomataceae
*, multigene phylogeny, new taxa, Northeast China, taxonomy

## Abstract

The *Rhodocybe*–*Clitopilus* clade of *Entolomataceae* exhibits high diversity in Northeast China, yet taxonomic studies on this group remain limited. Based on a field investigation conducted in this region (Jilin and Heilongjiang provinces), combined with morphological characteristics and multigene phylogenetic analyses (ITS, nrLSU, *rpb*2, and *tef*1-α), seven new species within four genera are proposed: *Clitopilus fraxinicola*, *Lulesia variabilicolor*, *Rhodocybe jilinensis*, *R. galericulata*, *R. eburneolutea*, *R. subnuciolens*, and *Rhodophana hongshiensis*. Phylogenetic analyses support the independent taxonomic status of these species within their respective genera. Detailed morphological descriptions, color photographs of basidiomata, and illustrations of microstructures are provided. Additionally, a dichotomous key to the known species of the *Rhodocybe*–*Clitopilus* clade in Northeast China is presented.

## Introduction

*Entolomataceae* Kotl. & Pouzar (*Agaricales*, *Basidiomycota*) encompasses species with diverse morphologies, such as tricholomatoid, gasteroid, collybioid, clitocyboid, omphalinoid, or pleurotoid (Co-David et al. 2009; Baroni and Matheny 2011; Noordeloos et al. 2012). Spore prints are pinkish-flesh; basidiospores are angular with pronounced or inconspicuous angles in profile view, have a smooth, longitudinally ridged surface, or possess undulate-pustulate ornamentations (Baroni 1981; Co-David et al. 2009; Noordeloos et al. 2012; Kluting et al. 2014; Bau 2018). Species within this family are widely distributed and are predominantly saprotrophic, with a few being parasitic or symbiotic (Sánchez-García and Matheny 2017; Koch and Herr 2021; Peng et al. 2021; Jian et al. 2025). In terms of symbiotic relationships, typical ectomycorrhizal lifestyles have been well documented in subg. *Entoloma* (Antibus et al. 1981; Loree et al. 1989; Agerer 1997). Furthermore, a distinctive symbiotic strategy is observed in the *E. clypeatum* (L.) P. Kumm. complex, which forms unique ectomycorrhiza-like structures with its host plants (Shishikura et al. 2021). They also hold significant economic value; for instance, *Clitopilus passeckerianus* (Pilát) Singer produces “pleuromutilin,” an antibiotic with a unique mechanism of action and efficacy against drug resistance (Bailey et al. 2016; Wu et al. 2019). Based on molecular phylogenetic studies, this family is divided into two core clades: the *Entoloma* clade and the *Rhodocybe*–*Clitopilus* clade (Co-David et al. 2009; Baroni and Matheny 2011). The latter includes five genera: *Rhodocybe* Maire, *Clitopilus* (Fr. ex Rabenh.) P. Kumm., *Clitopilopsis* Maire, *Lulesia* Singer, and *Rhodophana* Kühner (Kluting et al. 2014; Vizzini et al. 2023a).

The generic classification within the *Rhodocybe*–*Clitopilus* clade has undergone significant revision driven by molecular systematics (Co-David et al. 2009; Kluting et al. 2014). Morphologically, the genus *Clitopilus* is typically characterized by omphalinoid, clitocyboid, or pleurotoid basidiomata (Jian et al. 2020b). Its most distinctive feature is the basidiospores, which usually possess longitudinal ridges (appearing angular in polar view), although species with smooth spores are also nested within the genus (Moreno et al. 2007). Similarly, the traditional classification of *Rhodocybesensu lato* has been restructured based on molecular evidence. *Rhodophana* is recognized as an independent genus, morphologically distinguished from *Rhodocybe* s.str. by the presence of clamp connections (Kluting et al. 2014), while *Clitocella* is currently treated as a synonym of *Lulesia* (Varga et al. 2019; Vizzini et al. 2023a).

Previous research on *Entolomataceae* in Northeast China has predominantly focused on *Entoloma* (Fr.) P. Kumm. (Wang and Bau 2013; Ye 2022), leaving the *Rhodocybe*–*Clitopilus* clade largely unexplored in this region. Although the phylogenetic framework of this clade has been well established globally (Co-David et al. 2009; Kluting et al. 2014), species diversity and distribution in Northeast China remain poorly understood. Zhong (2019) and Jian (2020a) provided preliminary insights into these genera, notably identifying *rpb*2 and combined multigene datasets (nrLSU–*tef*1-α–*rpb*2–*atp*6) as effective phylogenetic markers. However, a comprehensive investigation of species from this specific region is still lacking. To address this gap, specimens from Jilin and Heilongjiang provinces were analyzed using integrative taxonomy. Seven new species are described herein, accompanied by detailed illustrations, to improve the understanding of *Entolomataceae* diversity in China.

## Materials and methods

### Samplings and morphological analyses

Systematic field investigations were conducted in Northeast China (Jilin and Heilongjiang provinces) from 2023 to 2025. Prior to collection, the ecological environment and vegetation type of each site were surveyed and photographed. During collection, detailed habitat data, substrate, and any distinctive odors of the basidiomata were recorded. Specimens at various developmental stages were collected to ensure morphological completeness. The colors of fresh basidiomata were documented using the RAL color system (Reichs-Ausschuss für Lieferbedingungen und Gütesicherung; https://www.ral-guetezeichen.de/). All voucher specimens are deposited in the Fungarium of Jilin Agricultural University (**FJAU**).

For microscopic examination, well-preserved specimens were selected. Free-hand sections were prepared under a stereomicroscope (Stemi 2000-C, Zeiss Co., Ltd., Oberkochen, Germany). Fresh material was mounted in water, while dried specimens were rehydrated in 5% KOH. Tissues were stained with 1% Congo red solution as needed to enhance structural contrast. The hymenophoral trama, cystidia, basidia, basidiospores, pileipellis, and stipitipellis were systematically examined. Melzer’s reagent was used to test for amyloid and dextrinoid reactions. Microscopic photography, feature documentation, and line drawing were performed simultaneously during observation.

Basidiospores were measured at 1000× magnification using an optical microscope and EP viewer V1.4 software (Olympus Co., Ltd., Tokyo, Japan). The notation [*n*/*m*/*p*] indicates that “*n*” basidiospores were measured from “*m*” basidiomata of “*p*” collections. Spore dimensions are presented as (minimum–) lower 90%–mean–upper 90% (–maximum) for length and similarly for width. The length/width ratio (*Q*) and mean (*Q_m_*) were calculated.

Scanning electron microscopy (SEM). Micromorphological features of basidiospores were examined using a CIQTEK SEM4000X field emission scanning electron microscope (FESEM) (Hefei, China) or a Hitachi Regulus 8100 FESEM (Tokyo, Japan). To restore spore turgidity and prevent collapse, dried lamellar fragments (approximately 2 mm^2^) were prepared following a modified protocol based on Kluting et al. (2014) and Nation (1983). Samples were first wetted in 95% ethanol (1 min), revived in 3% KOH (1 min), and rinsed twice in distilled water. Dehydration was performed through a graded ethanol series (10%, 30%, 50%, 70%, 80%, 90%, 95%) for 5 min each, followed by three changes of 100% anhydrous ethanol (10 min each). Chemical drying was then conducted using hexamethyldisilazane (HMDS) as an alternative to critical point drying. Dehydrated samples were treated with a 1:1 mixture of 100% ethanol and HMDS (15 min), followed by two changes of 100% HMDS (20 min each). The final volume of HMDS was allowed to evaporate slowly in a fume hood overnight. Dried specimens were mounted on aluminum stubs using double-sided conductive carbon tape, sputter-coated with gold (approximately 20 nm thickness), and examined at accelerating voltages ranging from 3 to 10 kV and working distances ranging from 5.2 to 12.6 mm.

### DNA extraction, PCR amplification, and sequencing

Total genomic DNA was extracted from dried basidioma tissues using the NuClean Plant Genomic DNA Kit (ComWin Biotech, CW0531M, Taizhou, China), following the manufacturer’s instructions. Polymerase chain reaction (PCR) amplification was performed with primer pairs ITS1F/ITS4 (White et al. 1990; Gardes and Bruns 1993), LR0R/LR5 (Vilgalys and Hester 1990; Rehner and Samuels 1994), b*rp*b2-6F/b*rp*b2-7.1R (Matheny 2005), and EF1-983F/EF1-1953R (Matheny et al. 2007). PCR amplifications were carried out in a 25 µL reaction volume containing 12.5 µL of 2× PrimeSTAR Mix Premix (B532061, Sangon Biotech, Shanghai, China), 1.0 µL of each primer (10 μM), 2.0 µL of template DNA, and 8.5 µL of ddH_2_O. The thermal cycling conditions consisted of initial denaturation at 94 °C for 5 min, followed by 35 cycles of denaturation at 94 °C for 50 s, annealing for 50 s (52 °C for ITS, 50 °C for nrLSU and *tef*1-α, and 55 °C for *rpb*2), and extension at 72 °C for 1 min, followed by final extension at 72 °C for 10 min. PCR products were visualized on 1% agarose gels and sent to Sangon Biotech (Shanghai, China) for sequencing. Primers and amplification conditions for each marker are listed in Table 1.

**Table 1.** Sequencing primers and the best annealing temperature for ITS, nrLSU, *rpb*2, and *tef*1-α.

Primer	Nucleotide sequence 5'–3'	PCR annealing temperature (°C)
ITS1F	CTTGGTCATTTAGAGGAAGTAA	52
ITS4	TCCTCCGCTTATTGATATGC	
LR0R	GTACCCGCTGAACTTAAGC	50
LR5	ATCCTGAGGGAAACTTC	
brpb2-6F	TGGGGYATGGTNTGYCCYGC	55
brpb2-7.1R	CCCATRGCYTGYTTMCCCATDGC	
EF1-983F	GCYCCYGGHCAYCGTGAYTTYAT	50
EF1-1953R	CCRGCRACRGTRTGTCTCAT	

### Phylogenetic analyses

Newly generated sequences were assembled and edited using Sequencher 4.1.4 (Gene Codes Corp., Ann Arbor, MI, USA) and deposited in GenBank (Sayers et al. 2025). To confirm the taxonomic identity of the new collections and select reference taxa for dataset construction, BLASTn searches were conducted using the algorithm of Altschul et al. (1997) against the GenBank and UNITE (Abarenkov et al. 2024) databases. All sequences used in the phylogenetic analysis are provided in Suppl. material 1. The four molecular markers (ITS, nrLSU, *rpb*2, and *tef*1-α) were aligned separately using the G-INS-i algorithm implemented in MAFFT v.7 (Katoh et al. 2019) via the online server (https://mafft.cbrc.jp/alignment/server/). To account for their heterogeneous evolutionary rates, the ITS region was partitioned into ITS1, 5.8S, and ITS2 for subsequent analyses. The alignments were manually refined and trimmed using MEGA7 (Kumar et al. 2016), and a concatenated multigene dataset was generated using PhyloSuite v2 (Zhao et al. 2025).

Maximum likelihood (ML) phylogenies were inferred using IQ-TREE 3 (Minh et al. 2020; Wong et al. 2025). The best-fit partition models were estimated using ModelFinder (Kalyaanamoorthy et al. 2017) implemented in IQ-TREE based on the Bayesian information criterion (BIC). Separate evolutionary models were estimated for each partition (ITS1, 5.8S, ITS2, nrLSU, *rpb*2, and *tef*1-α). Branch support was estimated using 1000 ultrafast bootstrap replicates (Hoang et al. 2018) and the Shimodaira–Hasegawa-like approximate likelihood-ratio test (SH-aLRT) (Guindon et al. 2010).

Bayesian inference (BI) was performed using MrBayes v.3.2.7 (Ronquist et al. 2012) implemented in PhyloSuite. A partitioned, mixed-model analysis was conducted with parameters unlinked across partitions. Two independent Markov chain Monte Carlo (MCMC) chains were run for 20 million generations, sampling every 1000 generations. The analysis was stopped when the average standard deviation of split frequencies (ASDSF) stabilized at 0.0108. Convergence was further confirmed using Tracer v.1.7 (Rambaut et al. 2018), which confirmed that all parameters had effective sample sizes (ESS) > 2000 and potential scale reduction factors (PSRF) approaching 1.0. The first 25% of the sampled trees were discarded as burn-in, and the remaining trees were used to calculate Bayesian posterior probabilities (PP). Phylogenetic trees were visualized and graphically optimized following the method described by Song and Bau (2024). In this study, *Clitocybe nebularis* and *Collybia tuberosa* were selected as outgroups. These genera are considered closely related to *Entolomataceae*; both belong to the tricholomatoid clade and have been used as outgroups in previous phylogenetic studies of this family (Co-David et al. 2009).

The genus abbreviations used in this study are as follows: *Clitopilus* = *C.*; *Clitopilopsis* = *Clp.*; *Rhodocybe* = *R.*; *Rhodophana* = *Rho.*; *Lulesia* = *L.*; *Entoloma* = *E.*

## Results

### Molecular phylogeny

**Figure 1.**
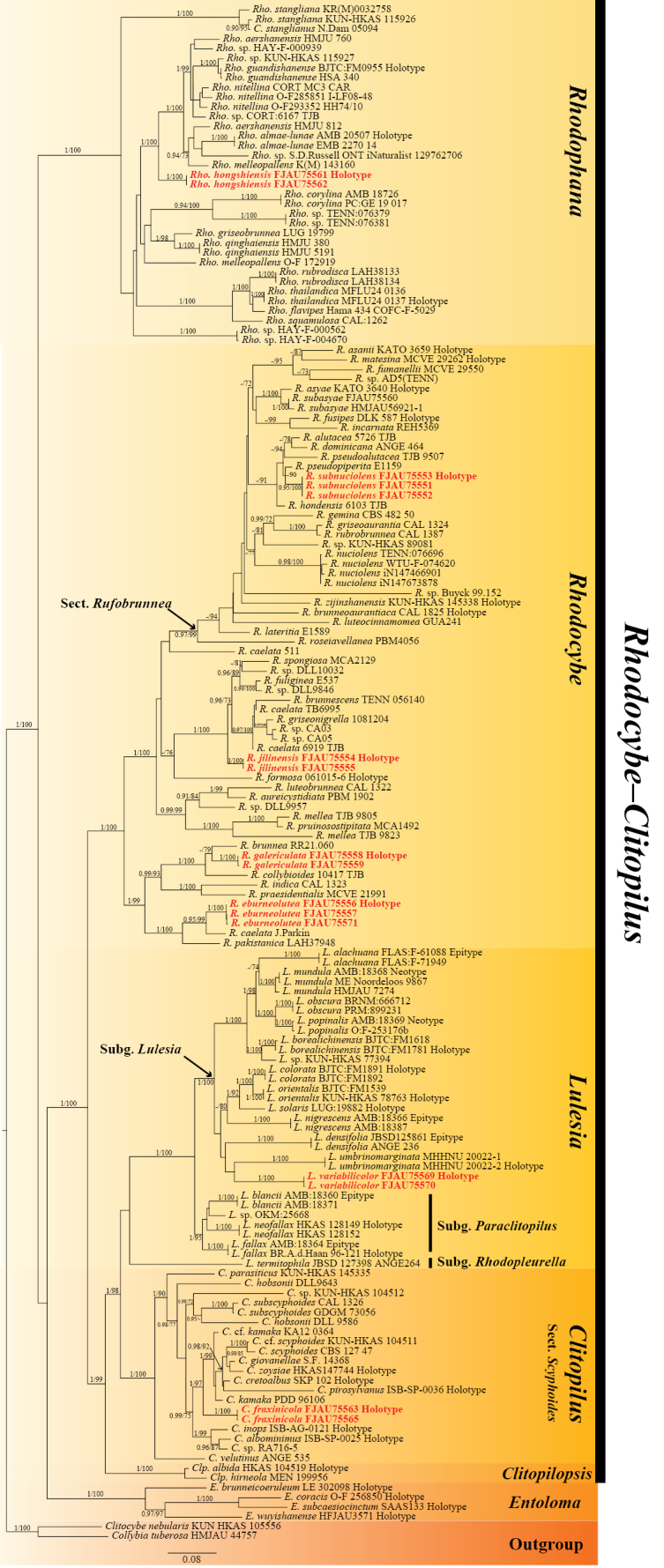
Maximum likelihood phylogeny of the *Rhodocybe*–*Clitopilus* clade (*Entolomataceae*) inferred from the combined multigene dataset (ITS, nrLSU, *rpb*2, and *tef*1-α). Maximum likelihood bootstrap support values ≥ 70% and Bayesian posterior probabilities ≥ 0.90 are indicated at the nodes (PP/BS). The seven newly proposed species are highlighted in bold red.

The maximum likelihood (ML) tree is presented in Fig. 1, while the Bayesian inference (BI) tree is provided in Suppl. material 2 due to their largely congruent topology. Bootstrap support values (BS) and Bayesian posterior probabilities (PP) are indicated at the nodes. Only support values of BS ≥ 70% and PP ≥ 0.90 are shown.

The combined multigene dataset (ITS, nrLSU, *rpb*2, and *tef*1-α) included 158 sequences with a total alignment length of 3670 columns (40.2% missing data). To address the variation in evolutionary rates, the ITS region was partitioned into ITS1, 5.8S, and ITS2. Statistics including sequence length, the proportion of variable sites, and parsimony-informative sites (PIS) were calculated for each partition: ITS1 (429 bp, 79.5% variable, 66.4% PIS); 5.8S (155 bp, 10.3% variable, 5.8% PIS); ITS2 (328 bp, 83.5% variable, 69.8% PIS); nrLSU (936 bp, 29.0% variable, 24.3% PIS); *rpb*2 (706 bp, 51.1% variable, 47.0% PIS); and *tef*1-α (1116 bp, 49.6% variable, 46.2% PIS). The best-fit models selected by BIC were TIM2+F+R4 (ITS1), TN+R2 (5.8S), TVM+F+R4 (ITS2), TIM3+I+R2 (nrLSU), GTR+F+I+R4 (*rpb*2), and TIM2e+I+G4 (*tef*1-α) for ML, and GTR+F+I+G4 (ITS1/ITS2/nrLSU/*rpb*2), K2P+G4 (5.8S), and SYM+I+G4 (*tef*1-α) for BI.

In the ML phylogenetic tree (Fig. 1), the seven newly proposed species are indicated in bold red. In the genus *Rhodophana*, *R. hongshiensis* formed a fully supported independent lineage (PP = 1, BS = 100). Within the genus *Rhodocybe*, four new species were identified: *R. subnuciolens* clustered within sect. *Rufobrunnea*, while *R. jilinensis*, *R. eburneolutea*, and *R. galericulata* all formed distinct branches. Notably, regarding *R. galericulata*, the ML analysis recovered it as sister to *R. brunnea* (specimen RR21.060; BS = 79), and the clade formed by these two species was sister to *R. collybioides* (specimen 10417 TJB; BS = 100, PP = 1.00). However, BI revealed a weakly supported grouping of *R. brunnea* and *R. collybioides* (PP = 0.66), with *R. galericulata* placed as a sister lineage to this pair (Suppl. material 2). Despite the topological discordance, the distinct phylogenetic position of *R. galericulata* as an independent species was consistent across both analyses. *Lulesia variabilicolor* clustered within subg. *Lulesia*. It was recovered as the sister taxon to *L. umbrinomarginata*; however, this relationship received weak support (BS = 66, PP = 0.51), suggesting that its precise phylogenetic placement within the subgenus requires further investigation. Finally, *Clitopilus fraxinicola* clustered within sect. *Scyphoides*, forming a strongly supported and independent branch (BS = 97, PP = 1).

### Molecular identification

BLASTn searches against the GenBank and UNITE databases confirmed the molecular distinctiveness of the seven new species. For *Rhodocybe subnuciolens*, BLAST searches of protein-coding genes confirmed its novelty, showing 97.6% identity to *R. pseudopiperita* (GQ289284) for *rpb*2 and 93.9% identity (KC816886) for *tef*1-α. For the other six species, ITS sequences confirmed significant genetic divergence from known taxa. *Rhodophana hongshiensis* shared only 86.5% identity with *Rhodophana* sp. (HAY-F-000939/SH0859573.10FU). *Rhodocybe jilinensis* showed 87.7% identity to *Rhodocybe* sp. (PQ847620). *Rhodocybe galericulata* showed 91.3% identity to *R. brunnea* (PV074066) in GenBank and 85.6% to *Clitopilus* sp. (SH0865870.10FU) in UNITE. *Rhodocybe eburneolutea* was most similar to *R. pakistanica* (NR_198629; 88.1% identity). *Lulesia variabilicolor* showed low identity (83.6%) to *L. popinalis* (FJ770400) and *Lulesia* sp. (SH0979876.10FU). *Clitopilus fraxinicola* showed 97.3% identity to *Clitopilus* sp. (OR860203), supporting its status as a distinct species.

### Taxonomy

#### Clitopilus fraxinicola

Taxon classificationFungiAgaricalesEntolomataceae

T. Bau & Tian Y. Zhang sp. nov.

9C12E3FC-95BF-56E0-A672-42AE8286C339

861194

Figs 2A, 2B, 5

##### Etymology.

The specific epithet “*fraxinicola*” is derived from Latin *fraxinus* (ash tree) and -*cola* (dweller), referring to its growth on decaying branches of the Manchurian ash (*Fraxinus mandshurica* Rupr.).

##### Holotype.

China. • Jilin Province, Jiaohe City, Qianjin Experimental Forest Farm, 16 June 2025, 43°57'29"N, 127°42'39"E, alt. 347 m, Tolgor Bau and Long-Hao Li LH2561602 (FJAU75563).

##### Diagnosis.

Basidiomata omphalinoid, signal white. Pileus centrally depressed; lamellae sparse; stipe central. Basidiospores subovoid to subfusiform with 6–8 longitudinal ridges. Gregarious on decaying branches of *Fraxinus mandshurica* in mixed forests.

##### Description.

Basidiomata omphalinoid. Pileus diameter 0.3–0.7 cm, initially hemispherical, later concave. When fresh, signal white (RAL 9003) to traffic white (RAL 9016); when dry, nearly white (RAL 1013). Pileus depressed at center, surface finely tomentose; margin entire, slightly incurved. Context thin, grayish white (RAL 9002); odor indistinct. Lamellae decurrent, white, sparse, unequally long. Stipe central, 0.7–2.1 cm long, 0.2 cm thick, subcylindrical, equal, solid, hyaline, signal white (RAL 9003), slightly curved in middle; surface smooth or finely tomentose; base with signal white (RAL 9003) mycelium.

##### Basidiospores.

[80/4/3] (5.9–)6.2–6.9–7.9(–8.3) × (3.2–)3.3–3.9–4.6(–4.9) µm, *Q* = 1.51–1.98, *Q_m_* = 1.75 (± 0.12), hyaline, subovoid to subfusiform or fusiform in profile and face view, obscurely angled in polar view with 6–8 facets produced by obscure longitudinal ridges (often indistinct under the light microscope), with minute transverse folds visible under SEM. Basidia 18–25 × 6–8 µm, clavate, 4 (2)-sterigmata. Cheilocystidia and pleurocystidia absent. Hymenophoral trama subregular, composed of subcylindrical hyphae 4–7 µm wide. Pileipellis a cutis, composed of interwoven, cylindrical, hyaline hyphae 5–8 µm in diameter, terminal cells 12–29 × 4–7 μm, subcylindrical, hyaline. Stipitipellis composed of repent, cylindrical hyphae 4–11 µm in diameter, interspersed with erect, hyaline hyphae 4–6 µm in diameter; terminal cells 15–39 × 5–8 μm, subcylindrical, hyaline, apex slightly acute or inflated. Clamp connections absent in all tissues.

**Figure 2.**
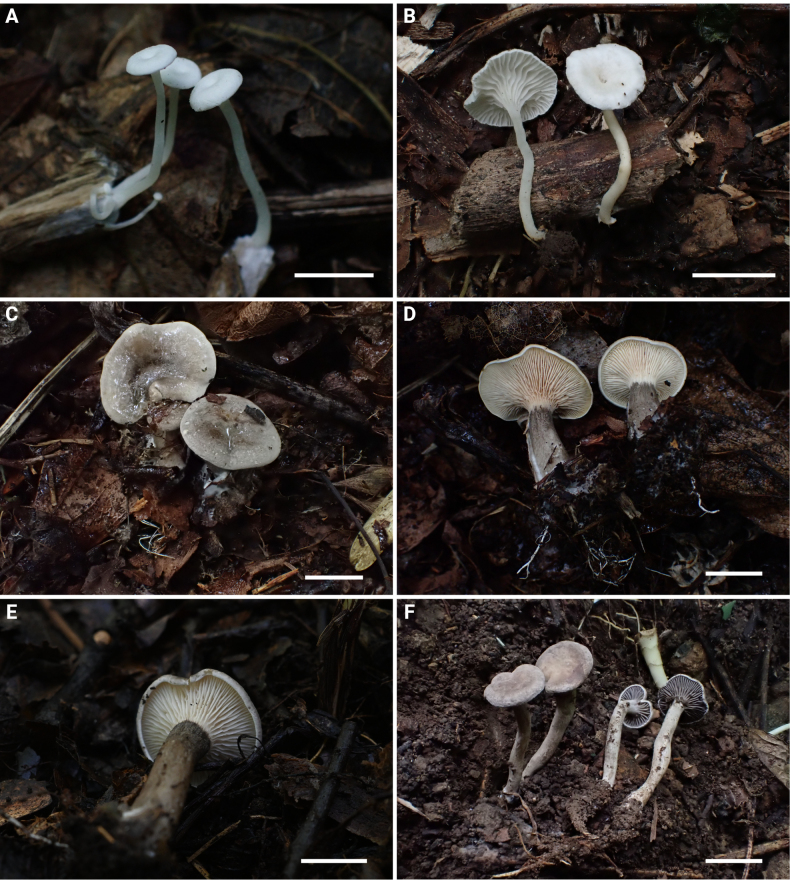
Basidiomata of the *Rhodocybe*–*Clitopilus* clade species. **A, B**. *Clitopilus fraxinicola* (**A**. FJAU75563; **B**. FJAU75565); **C–E**. *Lulesia variabilicolor* (**C, D**. FJAU75569; **E**. FJAU75570); **F**. *Rhodocybe jilinensis* (FJAU75554). Scale bars: 1 cm.

##### Habitat.

Gregarious on dead wood or branches of *Fraxinus mandshurica* in mixed forests during summer.

##### Distribution.

Currently known only from Jilin Province, China.

##### Additional specimens examined.

China. • Jilin Province, Jiaohe City, Qianjin Experimental Forest Farm, 29 June 2025, 43°57'29"N, 127°42'39"E, alt. 347 m, Hong Cheng C25062905 (FJAU75564), Tian-Yu Zhang ZTY25062909 (FJAU75565), Wei Sun SW25062912 (FJAU75567).

**Figure 3.**
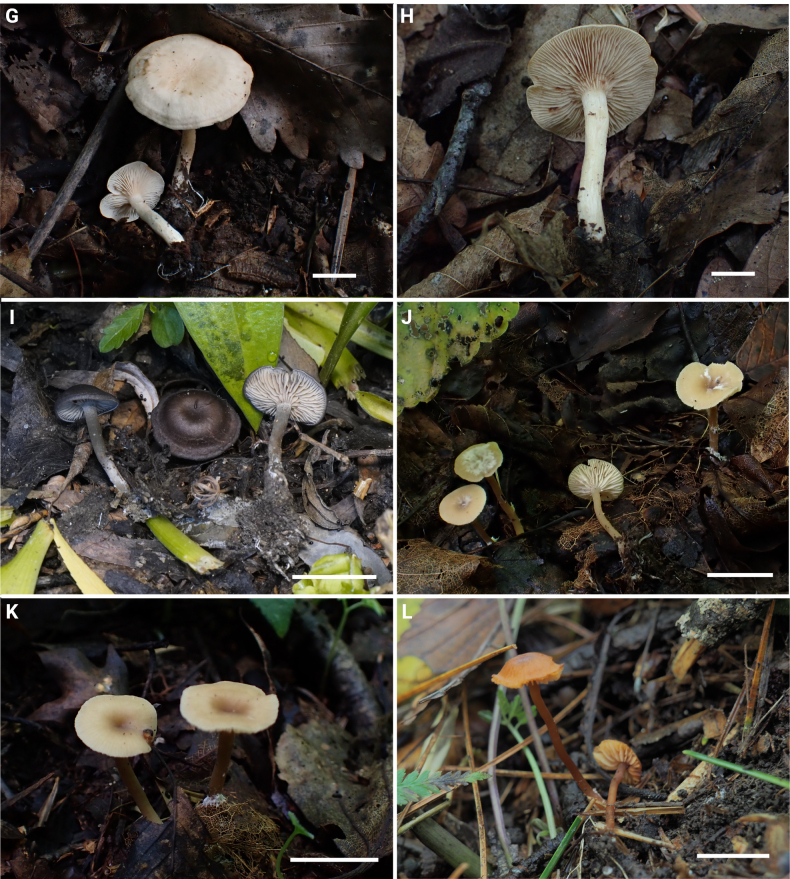
Basidiomata of the *Rhodocybe*–*Clitopilus* clade species. **G, H**. *Rhodocybe subnuciolens* (FJAU75553). **I**. *R. galericulata* (FJAU75558). **J, K**. *R. eburneolutea* (FJAU75556). **L**. *Rhodophana hongshiensis* (FJAU75561). Scale bars: 1 cm.

##### Notes.

Based on its morphology and phylogenetic position, this species belongs to *Clitopilus* sect. *Scyphoides*. Morphologically, the present species resembles *C. scyphoides* (Fr.) Singer, but the latter possesses a larger pileus (0.5–3 cm) and longer basidiospores (6.5–8.5 × 3.5–5.0 µm) and typically grows on soil, allowing differentiation (Noordeloos 1984; Singer 1986; Bas et al. 1988). While sharing small basidiomata with *C. subscyphoides* W.Q. Deng et al. from Guangdong, China, the latter species differs in occurring on soil and being distributed in subtropical regions (Deng et al. 2013; Jian et al. 2023). Furthermore, it is distinguished from its five phylogenetically close relatives by key characteristics: from *C. cretoalbus* Izhar et al. by the latter’s possession of polymorphic cheilocystidia (subcylindrical, lageniform to filiform) and soil habitat (Izhar et al. 2023); from *C. pirosylvanus* Asif et al. by the latter’s pale brown pileus with a deep depression and a smaller spore *Q* value (*Q_m_* = 1.4) (Asif et al. 2025); from *C. giovanellae* (Bres.) Singer by the latter’s smooth basidiospores (Moreno et al. 2007); and from both *C. zoysiae* Kun L. Yang et al. and *C. kamaka* J.A. Cooper by their pleurotoid basidiomata with an eccentric or reduced stipe, with the latter species also recorded from Oceania (Cooper 2014; Yang et al. 2025).

**Figure 4.**
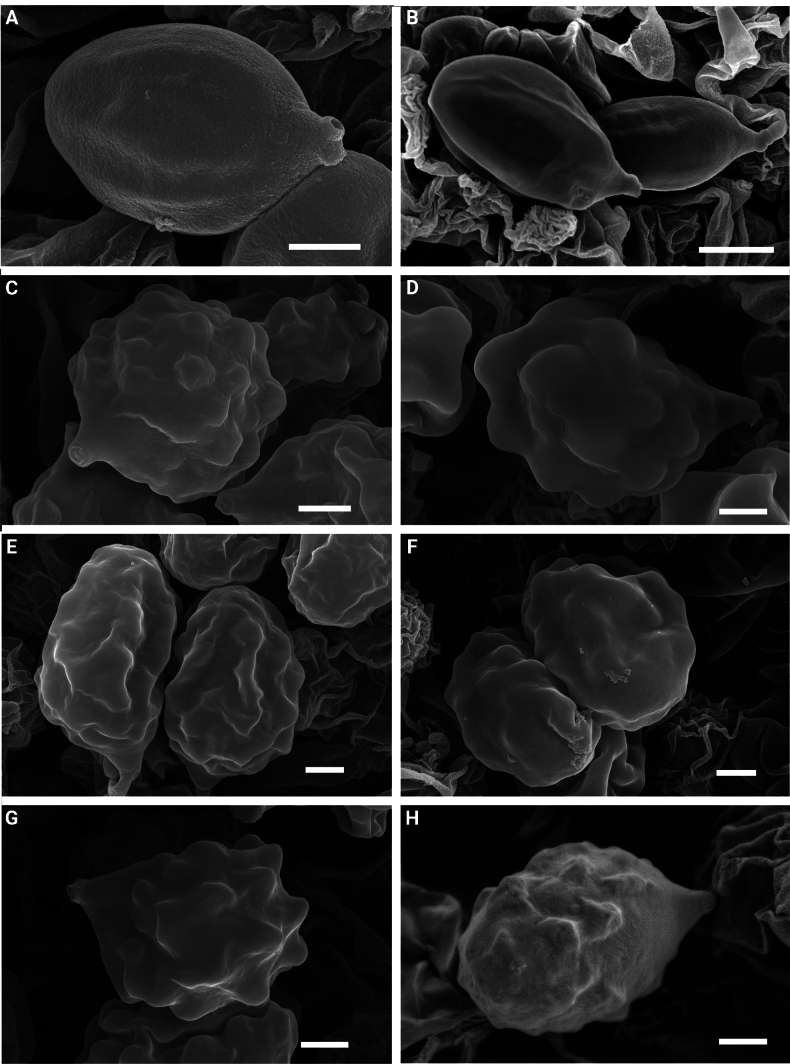
SEM of basidiospores in the *Rhodocybe*–*Clitopilus* clade. **A, B**. *Clitopilus fraxinicola* (FJAU75565); **C**. *Lulesia variabilicolor* (FJAU75569); **D**. *Rhodocybe jilinensis* (FJAU75554); **E**. *R. subnuciolens* (FJAU75553); **F**. *R. galericulata* (FJAU75558); **G**. *R. eburneolutea* (FJAU75556); **H**. *Rhodophana hongshiensis* (FJAU75561). Scale bars: 1 μm.

**Figure 5.**
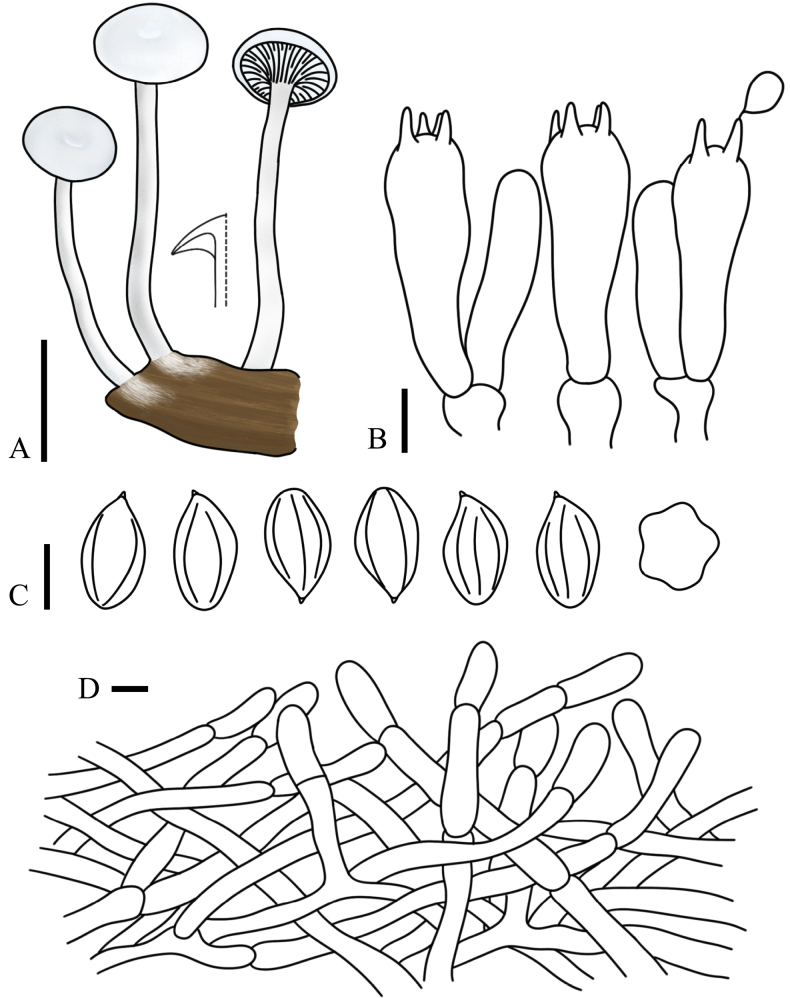
*Clitopilus fraxinicola* (FJAU75563, holotype). **A**. Basidiomata; **B**. Basidia; **C**. Basidiospores; **D**. Pileipellis. Scale bars: 1 cm (**A**); 5 µm (**B–D**).

#### Lulesia variabilicolor

Taxon classificationFungiAgaricalesEntolomataceae

T. Bau & Tian Y. Zhang sp. nov.

E721B6E9-690C-5E8A-87C2-891A786B2E27

861195

Figs 2C–E, 6

##### Etymology.

The epithet “*variabilicolor*” is a combination of Latin variabilis (variable) and color (color), alluding to the species’ highly variable pileus coloration.

##### Holotype.

China. • Jilin Province, Huadian City, Hongshi National Forest Park, 16 August 2025, 42°50'56"N, 127°07'12"E, alt. 498 m, Tian-Yu Zhang, ZTY2581617, (FJAU75569).

##### Diagnosis.

The pileus exhibits colors ranging from brownish gray to purple violet, is centrally slightly depressed. The stipe is central, cylindrical, and broadens downwards. The basidiospores are subglobose to broadly ellipsoid. The cheilocystidia are subfusiform with slightly curved apices. The hymenophoral trama is regular, and the pileipellis is a trichoderm.

##### Description.

Basidiomata collybioid. Pileus diameter 1.6–2.3 cm, initially hemispherical, later applanate. When fresh, center brown gray (RAL 7013), dark gray (RAL 7022), purple violet (RAL 4007) to blue-black (RAL 5004), margin yellow gray (RAL 7034), deep pigeon gray (RAL 7044), light gray (RAL 7035); when dry, light olive gray (RAL 7015). Pileus slightly depressed at center, covered in pruinose pubescence; margin entire, slightly incurved. Context thin, nearly white (RAL 1013), odor not distinctive. Lamellae decurrent, forking at stipe apex, gray white (RAL 9012), crowded, unequally long. Stipe central, 1.6–4.2 cm long, 0.7–1.1 cm thick, cylindrical, broadening downwards, hollow; apex concolorous with the lamellae, gradually turning to pearl light gray (RAL 9022) to pearl dark gray (RAL 9023), surface fibrillose, base slightly bulbous, with mycelium and rhizomorphs. Surfaces of dried basidiomata negative in KOH.

##### Basidiospores.

[60/3/2] (4.9–)5.1–5.4–5.8(–6.0) × (4.2–)4.3–4.6–4.9(–5.0) μm, *Q* = 1.06–1.28, *Q_m_* = 1.16 (± 0.06), hyaline, subglobose to broadly ellipsoid in profile view and face view, obscurely angled in polar view; ornamentation undulate-pustulate. Basidia 25–30 × 7–9 μm, clavate, 4(1, 2)-sterigmata. Cheilocystidia 23–35 × 5–8 μm, subfusiform, apex slightly curved, rare. Hymenophoral trama regular, composed of tightly arranged, cylindrical hyphae 2–5 µm wide. Pileipellis a trichoderm, composed of cylindrical hyphae 3–6 μm in diameter; terminal cells 17–37 × 6–11 μm, clavate, bullet-shaped, fusoid, with pale brown pigment. Stipitipellis a compact layer of repent, cylindrical hyphae 3–7 μm in diameter, with pale grayish-brown pigment. Clamp connections absent in all tissues.

##### Habitat.

Growing singly or scattered in mixed forests during the summer and autumn seasons.

**Figure 6.**
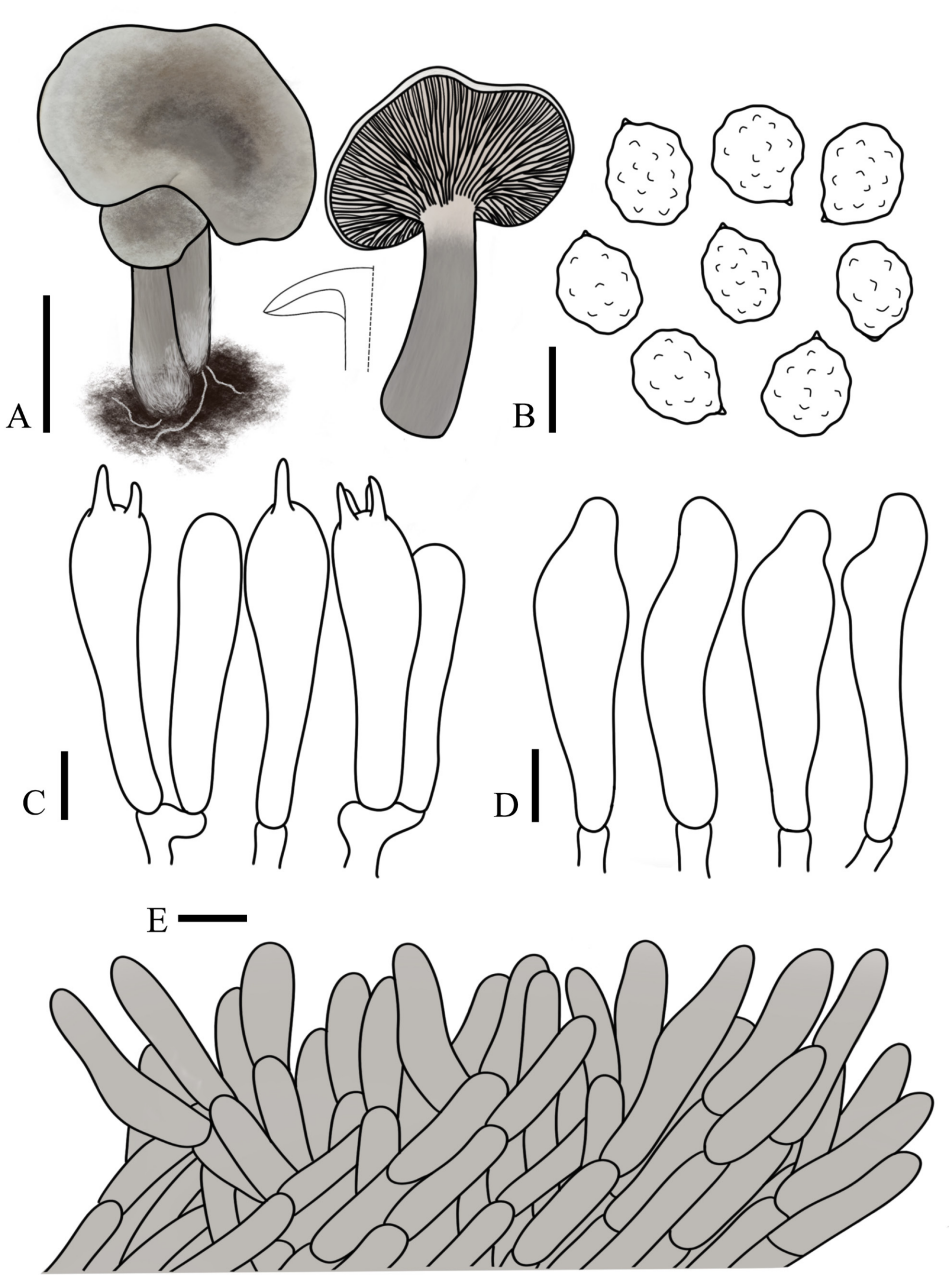
*Lulesia variabilicolor* (FJAU75569, holotype). **A**. Basidiomata; **B**. Basidiospores; **C**. Basidia; **D**. Cheilocystidia; **E**. Pileipellis. Scale bars: 1 cm (**A**); 5 µm (**B–E**).

##### Distribution.

Currently known only from Jilin Province, China.

##### Additional specimens examined.

China. • Jilin Province, Huadian City, Hongshi National Forest Park, 16 August 2024, 42°50'56"N, 127°07'12"E, alt. 498 m, Tolgor Bau and Ri-Hong Lin, LRH24081621, (FJAU75570).

##### Notes.

This species was initially discovered in 2024, with basidiomata exhibiting purple tones and occurring singly. It was recollected in 2025, during which the basidiomata displayed gray tones. Micromorphological and DNA sequencing data confirmed the conspecificity of both collections. Morphologically, characterized by its pileus with gray to purple tones and subglobose to broadly ellipsoid basidiospores, this new species fits well within subg. *Lulesia*, which presently comprises 10 species (Vizzini et al. 2023a). Although it shares a trichoderm pileipellis with *L. densifolia* (Singer) Singer, the latter is distinguished by its clitocyboid basidiomata, distinctly larger brown pileus (3–8 cm in diam.), and more slender stipe (2–8 × 0.5–0.8 cm) (Angelini and Contu 2012). While both the present species and *L. colorata* (L. Fan & N. Mao) T.J. Baroni et al. show considerable variation in pileus color, *L. colorata* is characterized by a white to gray-brown pileus, an irregular hymenophoral trama, absent cystidia, and a cutis pileipellis (Mao et al. 2022). Phylogenetically, the present species is a sister taxon to *L. umbrinomarginata* Y.Q. Xiao et al.; however, the latter is clearly distinguished by its deeply depressed pileus center, a reaction of the dried basidiomata to KOH, an irregular lamellar trama, and its subtropical distribution (Xiao et al. 2024). Compared to *L. nigrescens* (Maire) Vizzini et al., the latter is clearly distinguished by its typically pleurotoid basidiomata, a cutis pileipellis, and a positive red reaction to KOH on dried specimens (Vizzini et al. 2023b).

#### Rhodocybe jilinensis

Taxon classificationFungiAgaricalesEntolomataceae

T. Bau & Tian Y. Zhang sp. nov.

5C921ADE-ACB9-55D0-AFE6-EC254B5037C5

861197

Figs 2F, 7

##### Etymology.

The specific epithet “*jilinensis*” refers to Jilin Province, China, the origin of the type collection.

##### Holotype.

China. • Jilin Province, Ji’an City, Yushan Park, 24 July 2025, 41°08'18"N, 126°11'31"E, alt. 216 m, Long-Hao Li, LH2572405 (FJAU75554).

##### Diagnosis.

The basidiomata are omphalinoid. The pileus is light grayish brown to violet blue, centrally slightly depressed, with a velutinous surface. The lamellae are subdecurrent and white aluminium. The basidiospores are subellipsoid to subpyriform with strongly bumpy, undulate-pustulate ornamentation. The pseudocystidia are subcylindrical to subfusoid and non-dextrinoid.

##### Description.

Basidiomata omphalinoid. Pileus diameter 0.5–1.4 cm, initially hemispherical, later slightly applanate; when fresh violet blue (RAL 4009), dove gray (RAL 7032), platinum gray (RAL 7036); when dry chocolate brown (RAL 8017), light grayish brown (RAL 8028). Pileus slightly depressed at center, surface velutinous, non-striate; margin entire, slightly incurved. Context thin, deep brown (RAL 8011); odor indistinct or not distinctive. Lamellae subdecurrent, white aluminium (RAL 9006), pearl dark gray (RAL 9023), slightly crowded, unequally long. Stipe central, 1.4–2.9 cm long, 0.2–0.4 cm thick, cylindrical, equal or unequal, hollow, platinum gray (RAL 7036), slightly flexuous; surface fibrillose, base with signal white (RAL 9003) mycelium.

**Figure 7.**
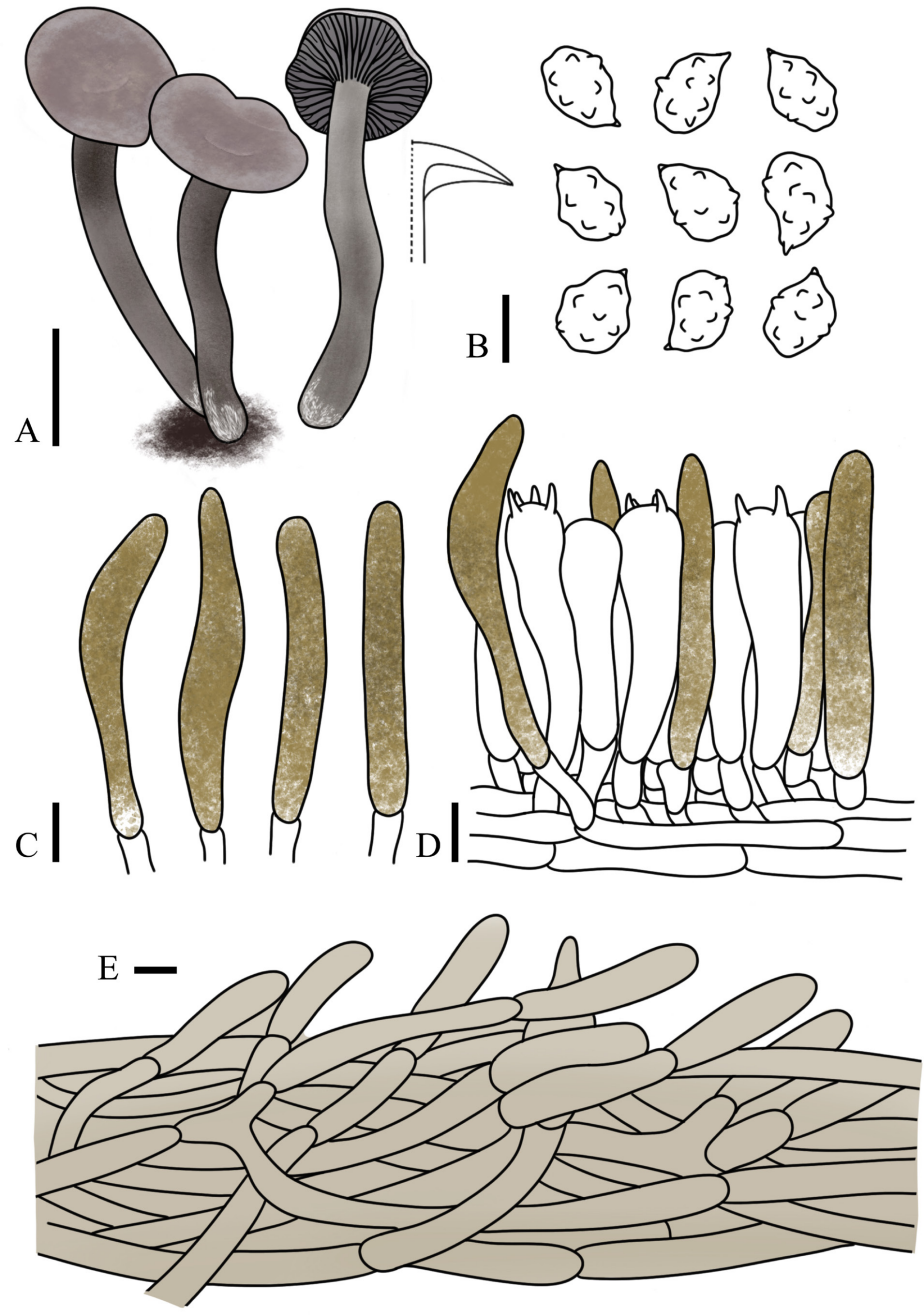
*Rhodocybe jilinensis* (FJAU75554, holotype). **A**. Basidiomata; **B**. Basidiospores; **C**. Cheilocystidia (pseudocystidia); **D**. Pleurocystidia (pseudocystidia) and basidia; **E**. Pileipellis. Scale bars: 1 cm (**A**); 5 µm (**B–E**).

##### Basidiospores.

[60/3/2] (5.9–)6.1–6.5–7.0(–7.2) × (4.6–)4.9–5.0–5.2(–5.6) μm, *Q* = 1.16–1.36, *Q_m_* = 1.29 (± 0.05), hyaline, subellipsoid to subpyriform in profile view, broadly ellipsoid in face view, distinctly angular in polar view, ornamentation strongly bumpy undulate-pustulate. Basidia 26–33 × 7–9 μm, clavate, 4(2)-sterigmata. Cheilocystidia (pseudocystidia) 27–43 × 4–7 μm, subcylindrical to subfusoid, with clay brown contents, non-dextrinoid. Pleurocystidia (pseudocystidia) similar to cheilocystidia in morphology, dimensions, and contents. Hymenophoral trama subregular, composed of cylindrical hyphae 5–7 μm in diameter, non-dextrinoid. Pileipellis a cutis with transitions to a trichoderm, composed of cylindrical hyphae 5–9 μm in diameter, with yellowish-brown to brown pigment, non-dextrinoid. Stipitipellis composed of repent, cylindrical hyphae 5–13 μm in diameter, with pale grayish-brown pigment. Clamp connections absent in all tissues.

##### Habitat.

Gregarious to scattered on the ground in mixed forests during summer.

##### Distribution.

Currently known only from Jilin Province, China.

##### Additional specimens examined.

China. • Jilin Province, Ji’an City, Yushan Park, 24 July 2025, 41°08'18"N, 126°11'31"E, alt. 216 m, Tolgor Bau and Yu Wang, WY2572413 (FJAU75555).

##### Notes.

*R. jilinensis* has abundant pseudocystidia and is classified in *Rhodocybe* sect. *Rhodocybe*. *R. caelata* (Fr.) Maire, the type species of the genus, shares a grayish pileus and abundant pseudocystidia with *R. jilinensis*. However, *R. caelata* is distinguished by its pileus, which often becomes concentrically areolate-rimose, and by its larger basidiospores (7.0–9.0 × 4.0–5.0 µm, *Q_m_* = 1.75), which are ellipsoid to oblong (Baroni 1981; Bas et al. 1988). *R. jilinensis* is macroscopically similar to *R. griseonigrella* (Vila, Contu, F. Caball. & Ribes) Vizzini et al. and can be easily confused since both species share small basidiomata with gray pileus and lamellae; however, the latter differs in subglobose basidiospores and a dextrinoid reaction in both the hymenium and pileipellis in Melzer’s reagent (Vila et al. 2009; Vizzini et al. 2016). *Rhodocybe brunnescens* T.J. Baroni & E. Horak is another comparable species, but it can be readily separated from *R. jilinensis* by its lamellae, which turn brown on bruising, and by its larger basidiospores (6.5–9.5 × 4.5–5.5 µm) (Baroni and Horak 1994).

Phylogenetically, *R. jilinensis* is closely related to *R. spongiosa* T.J. Baroni et al.; however, the latter is distinguished by its pleurotoid basidiomata, white coloration, and the absence of pigment in the pileipellis hyphae (Henkel et al. 2010). Compared to *R. fuliginea* E. Horak, the latter possesses a deeply umbilicate pileus, densely crowded lamellae, basidiospores that are distinctly rugulose to subangular, and acute-fusoid pseudocystidia (Horak 1979).

#### Rhodocybe subnuciolens

Taxon classificationFungiAgaricalesEntolomataceae

T. Bau & Tian Y. Zhang sp. nov.

67C6E162-D421-565F-B6F1-3D3D45F2AB1F

861196

Figs 3G, 3H, 8

##### Etymology.

The specific epithet “*subnuciolens*” refers to its morphological similarity to *R. nuciolens*.

##### Holotype.

China. • Jilin Province, Huadian City, Hongshi National Forest Park, 15 August 2025, 42°50'56"N, 127°07'12"E, alt. 498 m, Tian-Yu Zhang, ZTY2581517, (FJAU75553).

**Figure 8.**
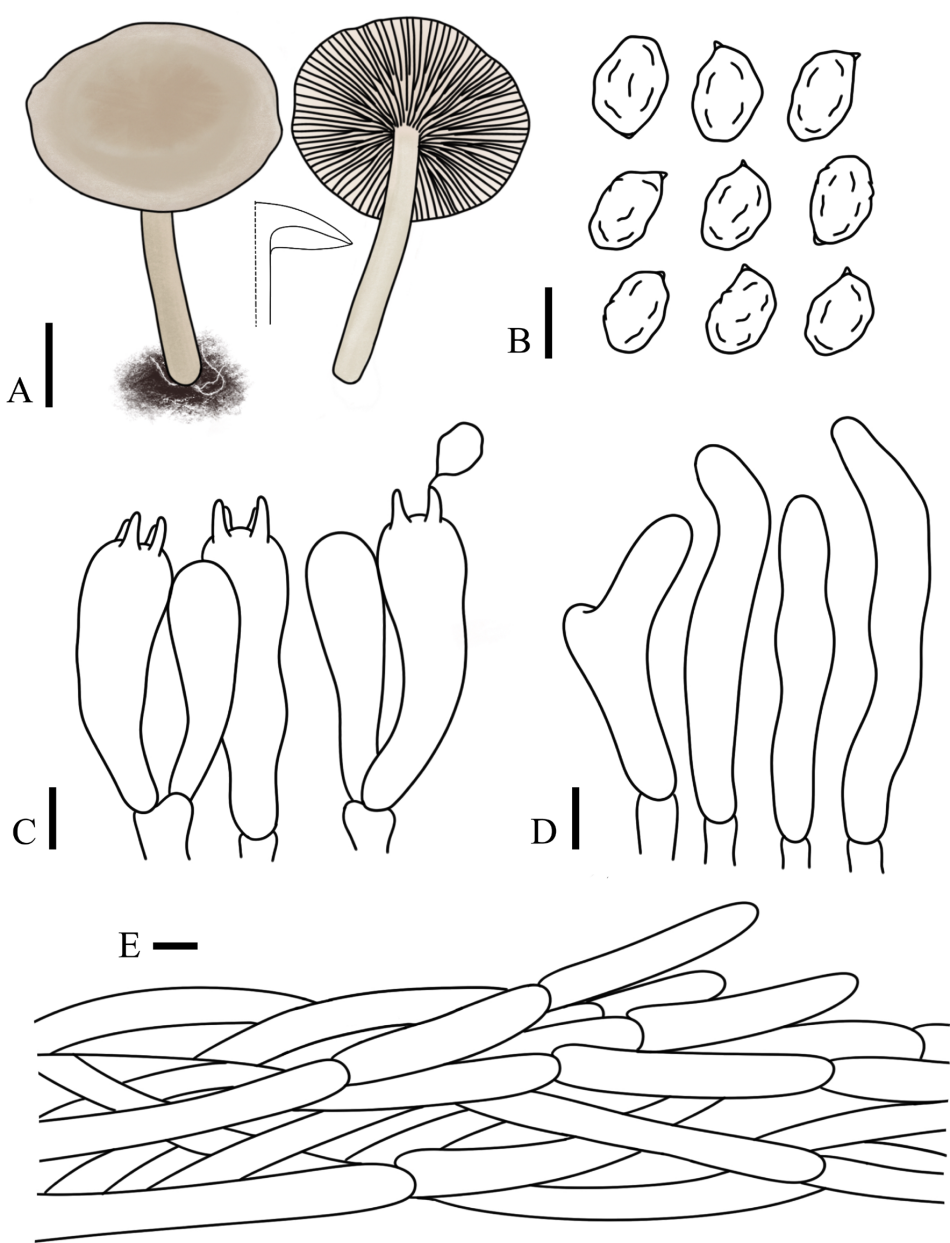
*Rhodocybe subnuciolens* (FJAU75553, holotype). **A**. Basidiomata; **B**. Basidiospores; **C**. Basidia; **D**. Cheilocystidia; **E**. Pileipellis. Scale bars: 1 cm (**A**); 5 µm (**B–E**).

##### Diagnosis.

Morphologically similar to *R. nuciolens*, but distinguished by its smaller and more slender basidiomata, smaller basidiospores, and more crowded lamellae.

##### Description.

Basidiomata collybioid. Pileus diameter 1.2–3.4 cm, initially convex, later applanate; when fresh grayish white (RAL 9012); when dry beige (RAL 1001). Pileus centrally slightly depressed or non-depressed; surface smooth, sometimes hygrophanous, non-striate; margin entire. Context slightly thin, sand yellow (RAL 1002); odor indistinct. Lamellae adnate to subdecurrent, off-white (RAL 1013), slightly crowded, unequally long. Stipe central, 1.7–2.9 cm long, 0.2–0.4 cm thick, cylindrical, equal, solid, grayish white (RAL 9012); surface smooth, basal signal white (RAL 9003), with mycelium and rhizomorphs.

##### Basidiospores.

[100/5/3] (4.8–)5.4–5.6–6.3(–6.5) × (3.3–)3.5–3.9–4.2(–4.5) μm, *Q* = 1.26–1.59, *Q_m_* = 1.45 (± 0.08), hyaline, subellipsoid or subamygdaliform in profile view, broadly ellipsoid in face view, minutely angular in polar view, undulate-pustulate in all views. Basidia 19–25 × 5–7 μm, clavate, 4(2)-sterigmata. Pseudocystidia absent. Cheilocystidia 17–33 × 3–5 μm, subcylindrical, often with bent or rarely branched apex, irregular, non-dextrinoid. Pleurocystidia absent. Hymenophoral trama regular, composed of cylindrical hyphae 7–12 μm in diameter, non-dextrinoid. Pileipellis a cutis, composed of thin-walled, hyaline, interwoven hyphae 6–9 μm in diameter, non-dextrinoid. Stipitipellis composed of repent, cylindrical, hyaline hyphae 5–8 μm in diameter. Clamp connections absent in all tissues.

##### Habitat.

Solitary or scattered on the ground in mixed forests during summer.

##### Distribution.

Currently known only from Jilin Province, China.

##### Additional specimens examined.

China. • Jilin Province, Huadian City, Hongshi National Forest Park, 16 August 2025, 42°50'56"N, 127°07'12"E, alt. 498 m, Tian-Yu Zhang, ZTY2581616, (FJAU75551); • Same location, 31 July 2025, Tian-Yu Zhang, ZTY2573118, (FJAU75552).

##### Notes.

*Rhodocybe subnuciolens* belongs to sect. *Rufobrunnea*. Compared with this species, *R. nuciolens* has larger basidiomata with a pileus diameter of 3.5–8 cm, more distant lamellae, larger basidiospores (5.5–8 × 4–5 μm), and is currently known only from North America (Murrill 1913). The basidiomata color of *R. subnuciolens* is close to that of *R. subasyae* T. Bau & Y.L. Sun, but the latter has a reddish tinge on the pileus, sinuate lamellae, and a two-layered pileipellis composed of short-cylindrical and inflated cylindrical hyphae. Furthermore, a significant genetic distance separates the two species, providing a clear basis for distinction (Sun and Bau 2023).

Phylogenetically, *R. subnuciolens* is a sister taxon to *R. pseudopiperita* T.J. Baroni & G.M. Gates, but the latter is distinguished by its lack of hymenial cystidia and its distribution in Oceania (Baroni and Gates 2006). The other three close relatives (*R. alutacea* Singer, *R. dominicana* T.J. Baroni & Angelini, and *R. pseudoalutacea* T.J. Baroni et al.) are all distributed in North America (Baroni et al. 2020). Among them, *R. alutacea* also possesses a pale pileus, but hymenial cystidia are absent; *R. dominicana* not only lacks hymenial cystidia but also has a two-layered pileipellis composed of cylindrical and subglobose hyphae; and *R. pseudoalutacea* has a brownish-orange pileus, lacks hymenial cystidia, and possesses caulocystidia.

#### Rhodocybe galericulata

Taxon classificationFungiAgaricalesEntolomataceae

T. Bau & T.Y. Zhang sp. nov.

AEAD8533-0B63-5D58-AB2D-4D186EFA0661

861198

Figs 3I, 9

##### Etymology.

The specific epithet “*galericulata*” is a Latin noun meaning “a small hat” or “cap,” referring to the characteristic bowler hat-like shape of the pileus, caused by its depressed center and raised surface.

**Figure 9.**
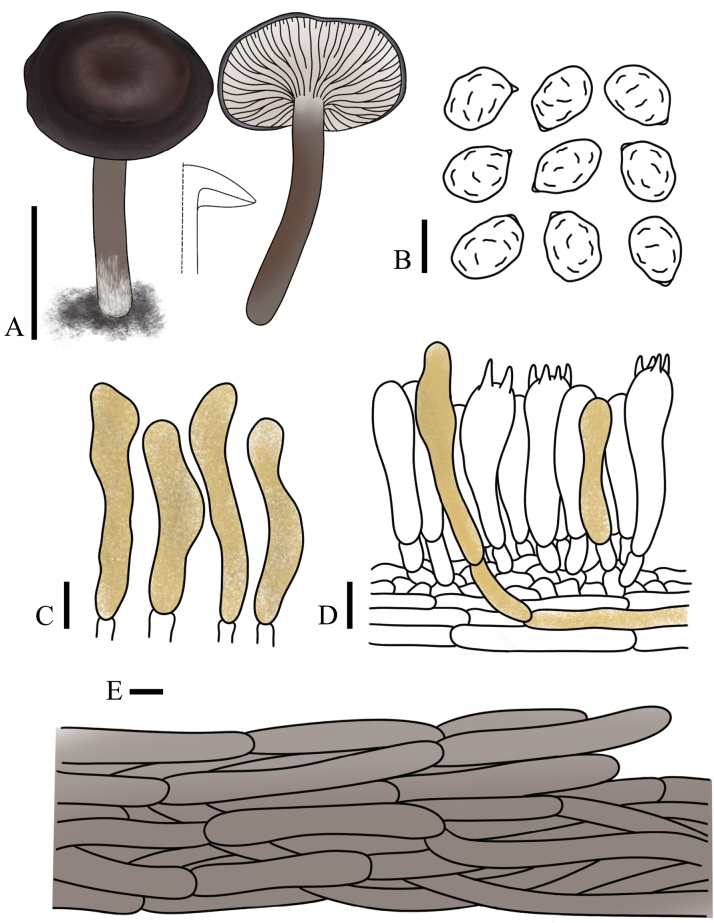
*Rhodocybe galericulata* (FJAU75558, holotype). **A**. Basidiomata; **B**. Basidiospores; **C**. Cheilocystidia (pseudocystidia); **D**. Pleurocystidia (pseudocystidia) and basidia; **E**. Pileipellis. Scale bars: 1 cm (**A**); 5 µm (**B–E**).

##### Holotype.

China. • Heilongjiang Province, Harbin City, Guli Park, 24 July 2023, 45°46'27"N, 124°41'47"E, alt. 128 m, Li-Yang Zhu, Z23072405 (FJAU75558).

##### Diagnosis.

Pileus chocolate brown to deep brown, centrally depressed with raised disc, resembling a bowler hat. Lamellae adnate to subdecurrent. Basidiospores subellipsoid to amygdaliform with undulate-pustulate ornamentation. Pseudocystidia clavate, subcylindrical to undulate in outline, with curry contents.

##### Description.

Basidiomata collybioid. Pileus diameter 0.6–1.0 cm, initially hemispherical to convex, later slightly applanate, eventually depressed at center, with area around the disc raised resembling a bowler hat; when fresh deep brown (RAL 8011), chestnut brown (RAL 8015), chocolate brown (RAL 8017); when dry blackish brown (RAL 8022), light grayish brown (RAL 8028). Pileus surface smooth; margin entire, slightly incurved. Context thin, soil brown (RAL 8003); odor indistinct. Lamellae adnate to subdecurrent, nearly white (RAL 1013), slightly crowded, unequally long. Stipe central, 1.2–1.7 cm long, 0.2 cm thick, cylindrical, equal, hollow, grayish brown (RAL 7013) to light brown (RAL 8025); surface smooth, base with signal white (RAL 9003) mycelium.

##### Basidiospores.

[80/4/2] (5.4–)5.6–6.1–6.8(–7.0) × (3.9–)4.0–4.5–4.9(–5.0) μm, *Q* = 1.22–1.56, *Q_m_* = 1.36 (± 0.08), hyaline, subellipsoid to amygdaliform in profile view, broadly ellipsoid in face view, obscurely angular in polar view, with undulate-pustulate ornamentation. Basidia 24–30 × 6–8 μm, clavate, 4(2)-sterigmata. Cheilocystidia (pseudocystidia) 20–45 × 3–8 μm, clavate, subcylindrical to undulate in outline, with curry contents, non-dextrinoid. Pleurocystidia (pseudocystidia) similar to cheilocystidia in morphology, dimensions, and contents. Hymenophoral trama subregular, composed of cylindrical hyphae 3–7 μm in diameter, non-dextrinoid. Pileipellis a cutis, composed of interwoven cylindrical hyphae 5–9 μm in diameter, with brown pigment, non-dextrinoid. Stipitipellis composed of repent, cylindrical hyphae 3–7 μm in diameter, with pale yellowish-brown pigment. Clamp connections absent in all tissues.

##### Habitat.

Gregarious on soil in broad-leaved forests during summer.

##### Distribution.

Currently known only from Heilongjiang Province, China.

##### Additional specimens examined.

China. • Heilongjiang Province, Harbin City, Guli Park, 24 July 2023, 45°46'27"N, 124°41'47"E, alt. 128 m, Wei-Nan Hou, H2307142 (FJAU75559).

##### Notes.

*R. galericulata* has pseudocystidia with curry contents and is morphologically classified within sect. *Rhodocybe*. While *R. galericulata* is macroscopically similar to *R. brunnea* Contu due to its brown pileus, the latter is distinguished by its larger basidiomata, a pileus ranging from pale yellowish to brown without a central depression, fusoid to lageniform pseudocystidia, and a gregarious habit on humus of *Cupressus sempervirens* L. (Contu 2006). *R. galericulata* also shares a brownish, centrally depressed pileus with *R. fuliginea* E. Horak; however, the latter differs in its densely crowded lamellae, a minutely velutinous to tomentose pileus surface, and more rounded, ovoid basidiospores (6.0–7.5 × 4.5–6.0 μm) (Horak 1979). Compared to its phylogenetically close relative *R. collybioides* Singer, the latter is distinguished by its slightly depressed pileus, solid stipe, and lanceolate-fusoid pseudocystidia with acute apices (Vrinda et al. 1999).

#### Rhodocybe eburneolutea

Taxon classificationFungiAgaricalesEntolomataceae

T. Bau & T.Y. Zhang sp. nov.

2D0442D1-DC2E-5133-A60A-B7846358C943

861206

Figs 3J, 3K, 10

##### Etymology.

The specific epithet “*eburneolutea*” refers to the ivory to yellowish coloration of the pileus, derived from the Latin ‘*eburneus*’ (ivory-colored) and ‘*luteus*’ (yellow).

**Figure 10.**
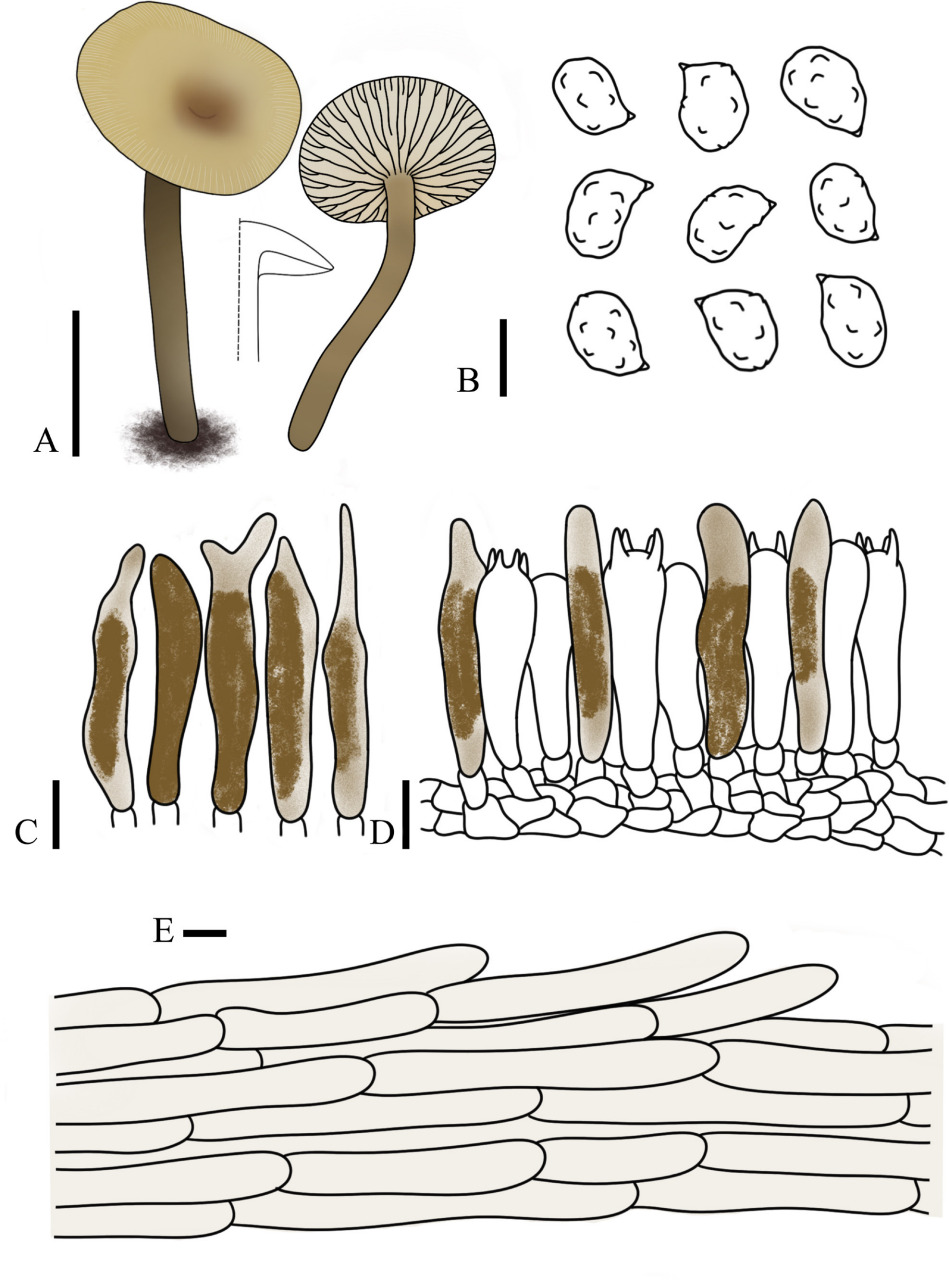
*Rhodocybe eburneolutea* (FJAU75556 Holotype). **A**. Basidiomata; **B**. Basidiospores; **C**. Cheilocystidia (pseudocystidia); **D**. Pleurocystidia (pseudocystidia) and basidia; **E**. Pileipellis. Scale bars: 1 cm **(A)**; 5 µm **(B–E)**.

##### Holotype.

China. • Jilin Province, Huadian City, Hongshi National Forest Park, 23 August 2024, 42°50'56"N, 127°07'12"E, alt. 498 m, Tolgor Bau and Xian-Yan Zhou, Y2482316 (FJAU75556).

##### Diagnosis.

The pileus is ivory to beige, centrally depressed, and brown beige. The pileus surface is smooth and translucent striate. The basidiospores are subellipsoid to obscurely angular, ornamented with bumpy undulate-pustulate. The pseudocystidia are abundant, predominantly subfusoid to rostrate, rarely branched, and non-dextrinoid. Both the hymenophoral trama and the pileipellis are non-dextrinoid.

##### Description.

Basidiomata clitocyboid. Pileus diameter 0.4–1.1 cm, initially hemispherical, later applanate, depressed at center; when fresh beige (RAL 1001) to ivory (RAL 1014), center brown beige (RAL 1011); when dry ochre brown (RAL 8001), clay brown (RAL 8003). Pileus surface smooth, translucent striate; margin entire. Context thin, off-white (RAL 1013); odor indistinct or not distinctive. Lamellae adnate to subdecurrent, off-white (RAL 1013) to bright ivory (RAL 1015), slightly crowded, unequally long, fragile. Stipe central, 0.7–1.4 cm long, 0.2 cm thick, cylindrical, equal, hollow, ivory (RAL 1014) to yellow ochre (RAL 1024), slightly flexuous; surface smooth.

##### Basidiospores.

[80/4/3] (5.3–)5.5–6.0–6.6(–6.7) × (3.9–)4.0–4.4–4.7(–5.0) μm, *Q* = 1.20–1.49, *Q_m_* = 1.37 (± 0.08), hyaline, subellipsoid and somewhat rounded angular in profile view, broadly ellipsoid in face view, obscurely angular in polar view, ornamentation strongly bumpy undulate-pustulate. Basidia 20–25 × 6–7 μm, clavate, 4(2)-sterigmata. Cheilocystidia (pseudocystidia) 23–41 × 4–7 μm, subclavate, narrowly lageniform, with a rarely branched apex, with golden pearl contents, non-dextrinoid. Pleurocystidia (pseudocystidia) 18–36 × 4–7 μm, flexuous, subcylindrical, subfusiform, with golden pearl contents, non-dextrinoid. Hymenophoral trama regular, composed of cylindrical hyphae 4–10 μm in diameter, non-dextrinoid. Pileipellis a cutis, composed of cylindrical hyphae 5–7 μm in diameter, with pale yellowish-brown pigment, non-dextrinoid. Stipitipellis composed of repent, cylindrical hyphae 3–5 μm in diameter, with yellowish-brown pigment. Clamp connections absent in all tissues.

##### Habitat.

Gregarious on the ground in mixed forests during summer.

##### Distribution.

Currently known only from Jilin Province, China.

##### Additional specimens examined.

China. • Jilin Province, Huadian City, Hongshi National Forest Park, 23 August 2024, 42°50'56"N, 127°07'12"E, alt. 498 m, Tian-Yu Zhang, ZTY2482307 (FJAU75557); • Same location, 15 September 2025, Yu-Fei Han, F25915027 (FJAU75571).

##### Notes.

Morphologically, *R. eburneolutea* is similar to *R. asyae* E. Sesli & Vizzini from Turkey, as both possess small, clitocyboid basidiomata with an ivory-colored pileus. However, the latter differs in lacking striations on the pileus surface, lacking pseudocystidia, and in its classification within sect. *Rufobrunnea*, allowing clear distinction (Sesli and Vizzini 2017). *R. eburneolutea* also shares a yellowish pileus and a slender stipe with *R. zijinshanensis* S.P. Jian & X. Chen from Nanjing, China, but *R. zijinshanensis* can be distinguished by its complete absence of hymenial cystidia and its distinct ecology of growing solitarily on rotten wood in broad-leaved forests (Jian et al. 2025).

Phylogenetically, *R. eburneolutea* is sister to *R. caelata* (Fr.) Maire (*rpb*2: KC816934). However, Kluting et al. (2014) have demonstrated that *R. caelata* as presently understood is polyphyletic with ambiguous species boundaries. This study reveals significant morphological differences between the new species and the polyphyletic *R. caelata*: the latter has more robust basidiomata, a grayish pileus that is velutinous and lacks translucent striations, larger basidiospores (7.0–9.0 × 4.0–5.0 µm, *Q_m_* = 1.75), and pseudocystidia that are mostly cylindrical to clavate (Baroni 1981; Bas et al. 1988). *R. eburneolutea* is also phylogenetically close to *R. pakistanica* Z. Khan & Khalid from Pakistan; nevertheless, *R. pakistanica* differs in its dark reddish-brown pileus, a stipe surface covered with white fibrils, and scarce pseudocystidia (Khan and Khalid 2024).

#### Rhodophana hongshiensis

Taxon classificationFungiAgaricalesEntolomataceae

T. Bau & Tian Y. Zhang sp. nov.

C0ED5D1A-4ADE-5E05-8A68-888577746604

861207

Figs 3L, 11

##### Etymology.

The specific epithet “*hongshiensis*” refers to Hongshi National Forest Park, the origin of the type collection.

##### Holotype.

China. • Jilin Province, Huadian City, Hongshi National Forest Park, 22 September 2024, 42°50'56"N, 127°07'12"E, alt. 498 m, Wei-Nan Hou, H2492218, (FJAU75561).

##### Diagnosis.

The pileus light orange-yellow to broom yellow, centrally depressed, smooth, with translucent striations at margin. Lamellae adnexed, slightly crowded. Stipe smooth. Basidiospores ellipsoid to amygdaliform with pronounced undulate-pustulate ornamentation.

##### Description.

Basidiomata collybioid. Pileus diameter 0.4–0.9 cm, initially hemispherical, later convex. When fresh, light orange-yellow (RAL 1028), broom yellow (RAL 1032); when dry, ochre-yellow (RAL 1024). Pileus depressed at center, surface smooth; margin with translucent striations, slightly recurved. Context thin, ivory (RAL 1014); odor indistinct. Lamellae adnexed, sandy yellow (RAL 1002), ventricose, slightly crowded, unequally long. Stipe central, 1.6–2.7 cm long, 0.2–0.3 cm thick, subcylindrical, soil-brown (RAL 8003), slightly curved, solid; surface smooth.

##### Basidiospores.

[40/2/2] (6.3–)6.4–6.7–7.5(–7.7) × (3.9–)4.1–4.3–4.6(–4.7) μm, *Q* = 1.35–1.77, *Q_m_* = 1.57 (± 0.10), hyaline, ellipsoid to amygdaliform in profile view, ellipsoid in face view, obscurely angular in polar view, with undulate-pustulate surface ornamentation. Basidia 22–28 × 8–9 μm, clavate, 4(2)-sterigmata. Cheilocystidia and pleurocystidia absent. Hymenophoral trama regular, composed of cylindrical hyphae 9–18 μm in diameter. Pileipellis a cutis, composed of cylindrical hyphae 9–16 μm in diameter, with pale yellowish pigment; terminal cells 38–77 × 12–23 μm, cylindrical, subfusoid. Stipitipellis composed of repent, cylindrical hyphae 6–11 μm in diameter, with yellowish-brown pigment. Clamp connections present in all tissues.

##### Habitat.

Solitary or scattered on the ground in mixed forests during summer.

##### Distribution.

Currently known only from Jilin Province, China.

##### Additional specimens examined.

China. • Jilin Province, Huadian City, Hongshi National Forest Park, 21 September 2024, 42°50'56"N, 127°07'12"E, alt. 498 m, Tolgor Bau and Tian-Yu Zhang, ZTY2492107, (FJAU75562).

##### Notes.

Morphologically, *Rho. hongshiensis* closely resembles *Rho. nitellina* (Fr.) Papetti in its orange-toned pileus with translucent striations. However, the latter can be distinguished by its significantly larger pileus (40–50 mm diam.) and larger basidiospores measuring 7.5–10 × 4.5–5.6 μm (Bas et al. 1988). Within the *Rho. nitellina* complex, a small-sized taxon, *Rho. nitellina* var. *minor* (Fr.) Papetti, has been documented. According to the taxonomic revision by Papetti, this entity was not elevated to species rank but was transferred from its previous placements in *Agaricus* and *Collybia* into *Rhodophana*, establishing the new combination *Rho. nitellina* var. *minor* (Fr.) Papetti. Compared to *Rho. hongshiensis*, *Rho. nitellina* var. *minor* possesses more robust basidiomata, a pileus with a more reddish-brown tinge, and grows gregariously on the ground in coniferous forests, allowing clear differentiation (Papetti 2014).

**Figure 11.**
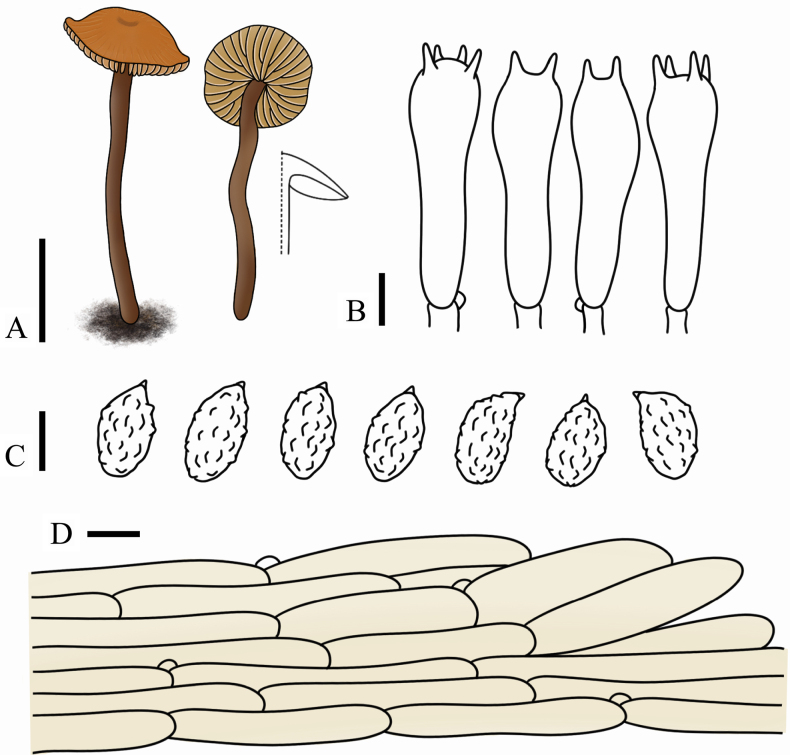
*Rhodophana hongshiensis* (FJAU75561, holotype). **A**. Basidiomata; **B**. Basidia; **C**. Basidiospores; **D**. Pileipellis. Scale bars: 1 cm (**A**); 5 µm (**B, C**); 10 µm (**D**).

*Rho. hongshiensis* is phylogenetically closely related to *Rho. almae*-*lunae* Papetti; however, the latter can be distinguished by its basidiospores with rounded pustules and its primary distribution in coniferous forests (on litter of *Pinus* or *Cedrus*) (Papetti 2024). Compared to *Rho. melleopallens* (P.D. Orton) Kluting et al., the latter has larger basidiomata and smaller basidiospores (4.5–7.0 × 3–4 μm, *Q* = 1.3–1.4) (Bas et al. 1988). *Rho. hongshiensis* and *Rho. aershanensis* J.Z. Xu from the Inner Mongolia Autonomous Region of China both possess small basidiomata. However, the latter has a reddish-brown pileus and smaller basidiospores (2.7–5.8 × 2.2–5.1 μm, *Q_m_* = 1.1) with slightly undulate-pustulate surfaces (Xu et al. 2023).

## Discussion

In the present study, phylogenetic relationships within the *Rhodocybe*–*Clitopilus* clade, specifically focusing on the genera *Clitopilus*, *Lulesia*, *Rhodocybe*, and *Rhodophana*, were evaluated by integrating newly collected Chinese materials. While guided by the phylogenetic backbone established by Kluting et al. (2014), the analyses utilized a distinct four-marker dataset (ITS, nrLSU, *rpb*2, and *tef*1-α). The analytical results are highly congruent with findings of previous studies (Baroni et al. 2020; Vizzini et al. 2023a), collectively confirming the relative stability of the internal phylogenetic structure within this clade and providing a robust framework for integrating morphology and systematics.

Following the phylogenetic framework, the integration of basidiospore ornamentation and ecological habitats provides crucial evidence for distinguishing taxa and reflecting their diversification within the *Rhodocybe*–*Clitopilus* clade. Morphologically, basidiospores exhibit significant intergeneric differences; for example, *C. fraxinicola* from *Clitopilus* sect. *Scyphoides* possesses distinct longitudinal ridges and minute transverse folds, which clearly distinguishes it from *Rhodocybe* species that are characterized by pronounced undulate-pustulate ornamentation (Co-David et al. 2009; Kluting et al. 2014). Beyond morphology, ecological traits exhibit profound plasticity, particularly within the genus *Clitopilus*. While many traditional species are strictly terricolous (soil-dwelling), the genus encompasses a remarkably diverse spectrum of nutritional strategies. For instance, species such as *C. zoysiae* and *C. parasiticus* S.P. Jian et al. (predominantly distributed in subtropical regions) demonstrate facultative or obligate parasitism on living host plants (Jian et al. 2025; Yang et al. 2025). Within this diverse ecological continuum, the new species *C. fraxinicola* occupies a distinct niche as a strict lignicolous saprotroph in temperate regions. This clear divergence in substrate preference—ranging from soil and living plant tissues to decaying woody debris—along with their diverse geographical distributions, highlights the remarkable ecological adaptability within the genus. Such lifestyle transitions and broad ecological plasticity have recently been supported by genomic evidence (Zhang et al. 2026). Building upon this evolutionary context, the findings emphasize that ecological traits and substrate preferences serve as highly valuable auxiliary characters for species recognition.

Within the genus *Lulesia*, the phylogenetic analysis supported the placement of *Lulesia variabilicolor* within subg. *Lulesia*, as its macroscopic morphology aligns with the circumscription of this subgenus provided by Vizzini et al. (2023a). Notably, this species shares a trichoderm-type pileipellis with the type species, *L. densifolia*, a feature that distinguishes them from other congeners. In the phylogenetic tree, *L. variabilicolor* clustered with *L. umbrinomarginata* and *L. densifolia*; however, this clade received low statistical support. Furthermore, certain characteristics of the basidiomata, specifically the negative reaction to KOH and the regular hymenophoral trama, correspond more closely to the definition of subg. *Paraclitopilus*. Based on these observations, *L. variabilicolor* may represent a transitional lineage bridging these two subgenera. Clarifying its definitive taxonomic status will require further comprehensive morphological, anatomical, and molecular phylogenetic studies.

*Rhodocybe* is traditionally subdivided into six sections within the *Rhodocybe*–*Clitopilus* clade. In the phylogenetic analyses, only sect. *Rufobrunnea* was resolved as a clearly monophyletic group, whereas the systematic boundaries of the remaining sections showed varying degrees of overlap, indicating that the current infrageneric classification system, based largely on macromorphology, requires revision. The type species, *R. caelata*, was recovered as polyphyletic, further confirming its complexity as a “species complex.” Furthermore, *R. galericulata* and *R. brunnea* were resolved as sister taxa. Since currently only a single ITS sequence of *R. brunnea* is available in the NCBI database (GenBank accession PV074066, originating from Switzerland; type locality: Italy), this identification was treated as provisional. Morphologically, differences in microscopic structures and habitats remain the key to distinguishing between these two species (Contu 2006).

Currently, the genus *Rhodophana* comprises a limited number of documented taxa. According to Index Fungorum (accessed 22 March 2026), this genus contains only 25 records, of which 17 represent formally described species. However, this limited number likely underestimates its true diversity. For instance, the type species, *R. nitellina*, constitutes a well-known species complex. Preliminary results presented by Dima et al. (2018) suggested the existence of 15 independent lineages within this complex based on a five-gene dataset. These lineages likely correspond to distinct species, indicating a high potential for discovering numerous new taxa. Consistent with this potential, the phylogenetic analysis recovered five *Rhodophana* species from China. Among them, the new species *R. hongshiensis* is morphologically distinct from its congeners, characterized by its diminutive, collybioid basidiomata and basidiospores with distinctly undulating pustulate ornamentation.

In conclusion, through an in-depth investigation of the *Rhodocybe*–*Clitopilus* clade, this study described and delineated seven new species spanning four genera, strongly supported by both multigene phylogenetic analyses and morphological evidence. The findings highlight the importance of Northeast China, a region sharing a temperate climate with much of Europe, as a reservoir of unrecognized fungal diversity. This work not only enriches the known species diversity of *Entolomataceae* in China but also provides crucial new data for understanding the evolution, ecological plasticity, and biogeographical connections of this important fungal group across the temperate regions of Eurasia and beyond.

### Key to the genera of the *Rhodocybe*–*Clitopilus* clade in Northeast China

**Table:** 

1	Basidiospores with distinct longitudinal ridges	*** Clitopilus ***
–	Basidiospores lacking longitudinal ridges	**2**
2	Basidiospores with obscure undulate-pustulate ornamentation (appearing nearly smooth under light microscope); lamellae long-decurrent	*** Lulesia ***
–	Basidiospores with pronounced undulate-pustulate ornamentation under light microscope; lamellae not long-decurrent	**3**
3	Hyphal clamp connections absent	*** Rhodocybe ***
–	Hyphal clamp connections present	*** Rhodophana ***

### Key to *Clitopilus* species in Northeast China

**Table:** 

1	Lignicolous (on *Fraxinus*); pileus white, < 1 cm; spores 6.2–7.9 µm	***C. fraxinicola***
–	Terricolous; pileus various; spores various	**2**
2	Pileus brownish-gray to gray; spores 8–12 µm, ridges 5–6	***C. brunneiceps***
–	Pileus white to whitish; spores (9.5–)10–13.5 µm long, ridges 5–7; pileus 2.5–7 cm	***C. prunulus***

### Key to *Lulesia* species in Northeast China

**Table:** 

1	Pileipellis a trichoderm; cheilocystidia present; pileus brownish-gray to purple-violet	***L. variabilicolor***
–	Pileipellis a cutis; cheilocystidia absent; pileus yellowish-gray to dark smoke gray with concentric zones	***L. mundula***

### Key to *Rhodocybe* species in Northeast China

**Table:** 

1	Pseudocystidia present (Sect. *Rhodocybe*)	**2**
–	Pseudocystidia absent (Sect. *Rufobrunnea*)	**4**
2	Pileus ivory to beige, surface translucent striate; pseudocystidia with golden contents	***R. eburneolutea***
–	Pileus brownish to violet blue, surface non-striate; pseudocystidia contents various	**3**
3	Pileus violet blue to platinum gray, surface velutinous; spores 6.1–7.0 µm	***R. jilinensis***
–	Pileus deep brown, bowler hat-shaped (depressed center, raised disc); spores 5.6–6.8 µm	***R. galericulata***
4	Pileus grayish white to beige; lamellae adnate to subdecurrent; spores 5.4–6.3 µm	***R. subnuciolens***
–	Pileus orange white to beige red; lamellae adnexed to sinuate; spores 5.4–6.8 µm	***R. subasyae***

### Key to *Rhodophana* species in Northern China

**Table:** 

1	Pileus light orange-yellow to yellow; lamellae adnexed; spores 6.4–7.5 µm long, ellipsoid to amygdaliform (*Q_m_* = 1.57)	***Rho. hongshiensis***
–	Pileus dark brown to reddish-brown; lamellae decurrent; spores 2.7–5.8 µm long, lacrymoid to subglobose (*Q_m_* = 1.1)	***Rho. aershanensis***

## Supplementary Material

XML Treatment for Clitopilus fraxinicola

XML Treatment for Lulesia variabilicolor

XML Treatment for Rhodocybe jilinensis

XML Treatment for Rhodocybe subnuciolens

XML Treatment for Rhodocybe galericulata

XML Treatment for Rhodocybe eburneolutea

XML Treatment for Rhodophana hongshiensis

## Additional information

### Artificial Intelligence (AI) use

The authors accept full responsibility for the content of the manuscript, including the disclosure of any use of AI.

No AI tools were used in the preparation of this manuscript.

### Author ORCIDs

T.-Y. Zhang https://orcid.org/0009-0005-5564-9629

T. Bau https://orcid.org/0000-0003-2461-9345

### Data availability

Newly generated sequences have been deposited in GenBank (https://www.ncbi.nlm.nih.gov), and all sequences used in the phylogenetic analysis are listed in Suppl. material 1. The multiple sequence alignments and phylogenetic tree files generated in this study are deposited in Zenodo (https://doi.org/10.5281/zenodo.17797902), while the Bayesian inference tree is provided in Suppl. material 2.

## Supplementary materials

Supplementary material 1 Sequeneces information used in the phylogenetic analysis Data type xlsx Explanation note A combined multi-marker dataset (ITS, nrLSU, rpb2, and tef1-α), comprising 158 taxa, was assembled for phylogenetic inference. https://binary.pensoft.net/file/1652588This dataset is made available under the Open Database License (http://opendatacommons.org/licenses/odbl/1.0/). The Open Database License (ODbL) is a license agreement intended to allow users to freely share, modify, and use this Dataset while maintaining this same freedom for others, provided that the original source and author(s) are credited. Tian-Yu Zhang, Tolgor Bau

Supplementary material 2 Bayesian inference tree Data type pdf Explanation note Bayesian posterior probabilities (PP) are indicated at the nodes. https://binary.pensoft.net/file/1652589This dataset is made available under the Open Database License (http://opendatacommons.org/licenses/odbl/1.0/). The Open Database License (ODbL) is a license agreement intended to allow users to freely share, modify, and use this Dataset while maintaining this same freedom for others, provided that the original source and author(s) are credited. Tian-Yu Zhang, Tolgor Bau
