# Supplementary figures and images for: Seven new species of the Rhodocybe– Clitopilus clade (Entolomataceae, Agaricales) from Northeast China
Source: MycoKeys. 2026 May 21;132:273–306. doi: 10.3897/mycokeys.132.181204 (PMC13220051; doi:10.3897/mycokeys.132.181204)

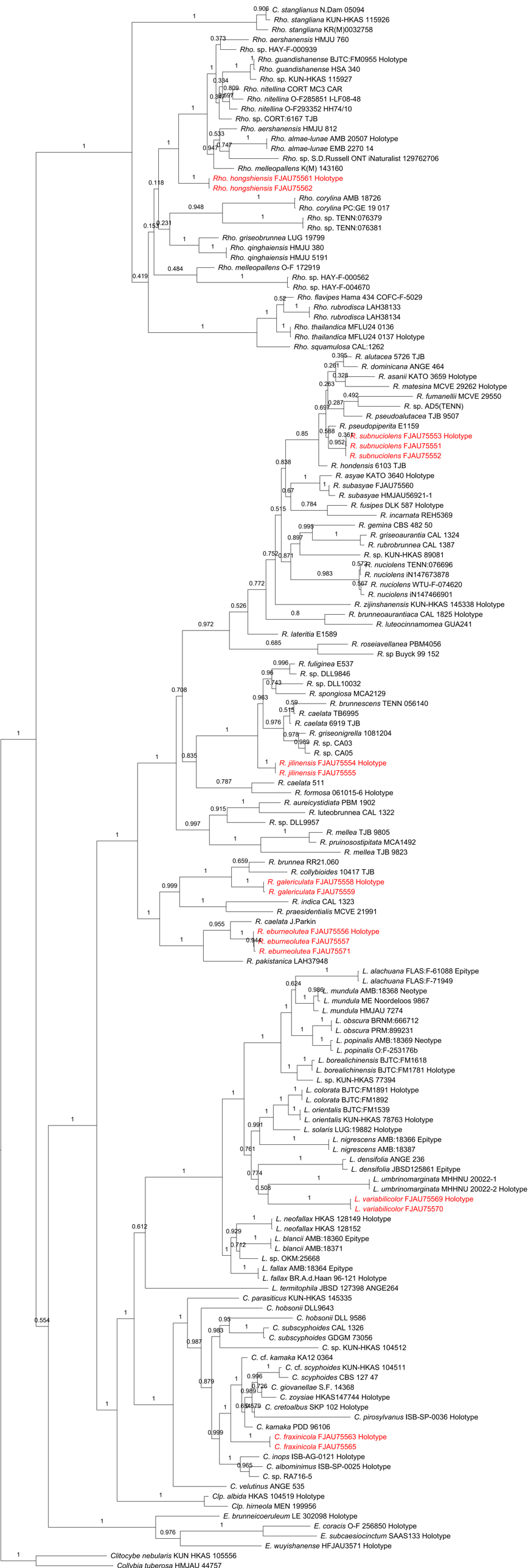

Supplement: Supplementary material 2 — Bayesian inference tree [file mycokeys-132-273-s002.pdf]
